# Intraocular pressure responses during maximal and submaximal handgrip strength tasks in primary open-angle glaucoma patients and healthy individuals

**DOI:** 10.7717/peerj.19845

**Published:** 2025-08-26

**Authors:** Cristina González-Hernández, Maria Dolores Morenas-Aguilar, Beatriz Redondo, Danica Janicijevic, María José López-Gómez, Jesus Vera, Amador García-Ramos

**Affiliations:** 1 Department of Physical Education and Sport, Faculty of Sport Sciences, Universidad de Granada, Granada, Andalucía, Spain; 2 CLARO (Clinical and Laboratory Applications of Research in Optometry) Laboratory, Department of Optics, Faculty of Sciences, Universidad de Granada, Granada, Andalucía, Spain; 3 Department of Radiology, Ningbo No. 2 Hospital, Ningbo, China; 4 Faculty of Sports Science, Ningbo University, Ningbo, China; 5 Department of Ophthalmology, Virgen de las Nieves University Hospital, Granada, Andalucía, Spain; 6 Department of Sports Sciences and Physical Conditioning, Faculty of Education, Universidad Católica de la Santísima Concepción, Concepción, Chile

**Keywords:** Health, Intraocular pressure, Ocular condition, Physical guidelines, Glaucoma

## Abstract

**Background:**

This study aimed to compare intraocular pressure (IOP) responses during isometric handgrip strength tasks between primary open-angle glaucoma patients and healthy individuals.

**Methods:**

Forty older adults participated: 21 glaucoma patients and 19 controls. Participants randomly performed four trials at two intensities, maximal and submaximal (at 50% of self-perceived maximal effort), with two trials per hand (one for the left eye and one for the right eye). IOP was measured immediately before exercise, during exercise, and 5 seconds post-recovery. A four-factor mixed ANOVA was used to analyze IOP responses, with task intensity and time of measurement as within-subject factors, and group (glaucoma vs. control) and sex as the between-subjects factors.

**Results:**

IOP responses were comparable between glaucoma patients and healthy individuals, as the main effect of group (*p* = 0.117) and its interactions did not reach statistical significance. The main effect of time reached statistical significance (*p* = 0.016) due to greater IOP values during the task (17.3 ± 3.7 mmHg) compared to pre-exercise (16.3 ± 3.2 mmHg: *p* < 0.001), but post-exercise IOP (16.9 ± 4.1 mmHg) was not significantly different from pre-exercise (*p* = 0.334) or during exercise (*p* = 0.727). Greater IOP values were observed for the maximal task compared to the submaximal task (*p* = 0.001), with no differences between men and women (*p* = 0.699).

**Conclusion:**

Submaximal isometric strength training is a safe option for glaucoma patients treated with hypotensive eye drops.

## Introduction

Glaucoma, one of the leading causes of irreversible blindness worldwide, has seen a rising prevalence over the years, with an estimated 76 million patients (Boland et al., 2013). Primary open-angle glaucoma (POAG) is the most common form of glaucoma, particularly in older adults, and often progresses silently until significant visual field loss has occurred. A critical factor in the progression of glaucoma is intraocular pressure (IOP), with the only proven strategy for effectively managing glaucoma being the reduction and stabilization of IOP levels (Boland et al., 2013). The standard treatment for POAG includes topical medications (mainly prostaglandin analogs and beta-blockers), laser therapy or surgical interventions. If left untreated or inadequately managed, POAG can lead to progressive optic nerve damage, irreversible peripheral vision loss, and eventual blindness. Both nonmodifiable factors, such as ageing, genetics, and ethnicity, and modifiable factors, including smoking, diet, pollution, and physical activity, can influence IOP levels (Hecht et al., 2017). Among these, physical exercise has received significant scientific attention due to its numerous health benefits, such as reducing the risk of cancer and fall-related injuries, and improving bone, cardiovascular, and brain health (Piercy et al., 2018). However, it is essential to approach physical exercise with caution in glaucoma patients, as it can induce acute increases in IOP, potentially exacerbating the condition (Zhu et al., 2018). Therefore, understanding the relationship between physical exercise and IOP is crucial for the safe management of this ocular condition.

Previous studies have demonstrated that the IOP response to physical exercise depends on various factors, including the type of exercise (*e.g.*, endurance or resistance training) (Hackett et al., 2024), exercise intensity (Conte & Scarpi, 2014), the individual’s fitness level (Prum et al., 2016), body position (Lara et al., 2023), breathing patterns during exercise (Sun et al., 2020), underlying medical conditions (Hecht et al., 2017), concurrent mental effort (Vera et al., 2018a; Vera et al., 2018b), and caffeine intake prior to exercise (Yoon & Danesh-Meyer, 2019). For instance, low-intensity aerobic exercise tends to lower IOP (Ma et al., 2022), whereas high-intensity strength training, such as lifting heavy loads or performing intense isometric contractions, has been shown to increase IOP (Vera et al., 2020). These studies provide valuable insights that can help guide exercise recommendations for glaucoma patients and those at risk. However, a limitation is that much of the existing research has primarily focused on healthy young individuals, and recommendations have been extrapolated to glaucoma patients. There is substantial evidence that glaucoma patients have an altered outflow facility of the eye, and pharmacological and surgical interventions are targeted to regulate this function (Stamer & Acott, 2012; Zhu et al., 2018; Vera et al., 2020). However, the impact of pharmacological interventions on aqueous humor regulation is dependent on the type of medication prescribed, with the most common options being targeted to regulate IOP levels by increasing the outflow (prostaglandin analogues) or reducing aqueous humor production (beta-blockers) (Stamer & Acott, 2012). Remarkably, untreated glaucoma patients have showed a higher outflow resistance to provocative tests, causing a significant increase of IOP levels (Pérez-Castilla et al., 2021). This highlights the importance of understanding whether the acute IOP response to physical exercise differs between glaucoma patients and healthy individuals. Addressing this gap is crucial for developing tailored exercise guidelines for glaucoma patients to ensure their safety and the effective management of their condition.

Isometric tasks, a type of exercise in which the muscles generate force while maintaining a constant position, have been shown to promote more pronounced fluctuations in IOP compared to dynamic tasks (Vera et al., 2017; Vera et al., 2018a; Vera et al., 2018b; Vera et al., 2019). Previous studies have found increments in IOP ranging from 0.7 to 28.7 mmHg during the execution of isometric tasks (Hecht et al., 2017; Vera et al., 2018a; Vera et al., 2018b; Vera et al., 2020). The large variability in the magnitude of IOP changes reported could be attributed to differences in the specific exercises performed and the intensity of the contractions. For instance, handgrip exercises have been shown to produce lower changes in IOP (0.7 to 3.6 mmHg) compared to other forms of isometric exercise such as squats (7.5 to 8.8 mmHg), biceps curl (3.1 to 9.4 mmHg), and bench press (13 to 28 mmHg) (Hackett et al., 2024). Furthermore, the increase in IOP is more pronounced when exercises are performed against greater external loads (Vera et al., 2017). Given that strength training is crucial for enhancing physical fitness, and high fitness levels seems to be a protective factor against the development of glaucoma (Meier et al., 2018; Madjedi et al., 2023), there is a pressing need to identify safe strength training methods for glaucoma patients.

The present study was designed to provide deeper insights into the selective responses of IOP to physical exercise in glaucoma patients compared to healthy individuals. Specifically, the primary objective was to compare IOP responses during maximal and submaximal isometric handgrip tasks between POAG patients and healthy individuals matched by age and sex. We hypothesized that POAG patients and healthy controls will exhibit a similar IOP response, since the POAG patients included in this study are medically treated with hypotensive eye drops targeted to regulate the outflow facility of the aqueous humor (Stamer & Acott, 2012; Zhu et al., 2018; Vera et al., 2020). Additionally, we hypothesized comparable changes in IOP for both men and women as the efforts were adjusted relative to their maximal capacities (Pérez-Castilla et al., 2021), whereas we expected greater increments in IOP during the maximal task compared to the submaximal task (Conte & Scarpi, 2014; Vera et al., 2017).

## Materials & Methods

### Subjects

Forty Caucasian older adults volunteered to participate in this study (Table 1). Among them, 21 participants (13 women and eight men) had been clinically diagnosed with POAG, while 19 participants (12 women and seven men) had no history or diagnosis of glaucoma. POAG diagnosis followed the objective criteria recommended by the American Academy of Ophthalmology (Prum et al., 2016), including glaucomatous optic nerve head changes and visual field defects consistent with glaucoma, after excluding of other possible causes. All patients were under stable topical treatment: 12 with prostaglandin analogs alone and nine with a combination of prostaglandin analogs and beta-blockers.

**Table 1:** Anthropometric characteristics of the study sample.

	Glaucoma patients			Healthy individuals		*p*-value
	Men	Women		Men	Women	
Sample size	8	13		7	12	
Age (years)	65.5 ± 6.3	70.9 ± 8.1		71.3 ± 6.8	68.2 ± 5.0	0.870
Body mass (kg)	81.5 ± 14.0	64.9 ± 9.5		80.4 ± 15.9	65.5 ± 6.3	0.861
Body height (cm)	169 ± 6	157 ± 6		171 ± 6	158 ± 5	0.718
BMI (kg/m^2^)	28.6 ± 4.7	26.5 ± 4.2		27.2 ± 3.5	26.3 ± 2.5	0.651

**Notes.**

BMI body mass index

Mean ± standard deviation.

Participants were recruited based on the following inclusion criteria: (a) age ≥ 60 years; (b) ability to perform supervised physical exercise independently; (c) no history of glaucoma surgery or laser intervention; (d) absence of systemic diseases or conditions contraindicating exercise participation (*e.g.*, unstable cardiovascular disease, uncontrolled hypertension, musculoskeletal disorders). Exclusion criteria included: (a) secondary forms of glaucoma; (b) current use of systemic medications known to affect IOP; and (c) cognitive impairment affecting understanding or compliance. All participants provided written informed consent. The study was conducted in accordance with the Declaration of Helsinki and was approved by the Regional Ethical Committee of Biomedical Research (IRB approval 1961-N-22).

### Design

A cross-over study design was used to examine the influence of performing maximal and submaximal isometric handgrip tasks on IOP behavior in POAG patients and healthy individuals. Participants attended the laboratory for a single session during which they performed eight trials in total. These included four trials of the maximal task and four trials of the submaximal task. For each task intensity, participants performed two trials with the right hand and two trials with the left hand. In half of the trials for each hand, the left eye was measured, while in the other half, the right eye was measured. To simplify the results and ensure more robust data, the statistical analyses used the average value of the four attempts performed for the same task intensity, considering measurements from both hands and both eyes. IOP was measured at three time points: immediately before exercise, during exercise, and after 5 s of recovery. The eight different trials were performed in a randomized order to minimize the possible effects of fatigue on the study’s findings. Data collection was carried out over a 6-month period, from January to June 2024. All participants were tested under similar environmental conditions (approximately 22 °C and 60% humidity) and were not allowed to drink or eat during the course of the experiment.

### Procedures

At the beginning of the session, participants’ age, body mass, and height were recorded. Subsequently, as part of the warm-up, participants performed several familiarization trials with both hands, using both maximal effort and submaximal effort (approximately 50% of their maximum). Performance feedback was provided following the maximal and submaximal attempts to help participants learn how to produce approximately 50% of their maximal force in the submaximal trials. After completing the familiarization stage, participants were given a 5-minute rest period before the experimental trials were initiated.

Participants performed the isometric handgrip strength task in a standing position, with the arm fully extended, ensuring the dynamometer only contacted the hand being tested (Ruiz et al., 2006). Participants were instructed to exert maximal or submaximal force from the start of the task, which lasted approximately 5 s, the duration needed for an IOP measurement. For the submaximal task, participants aimed to exert 50% of their maximal force. A two-minute rest period was provided between successive trials. Verbal encouragement was given immediately before each trial to ensure participants met the criteria for maximal or half of their maximum isometric strength. IOP was recorded in a standing position immediately before the handgrip task (baseline measurement), during the task (intra-effort measurement), and 5 s after completing the task (post-effort measurement).

Handgrip strength was assessed using a validated TKK dynamometer (TKK 5001 Grip-D; Takey, Tokyo, Japan) (Garber et al., 2011). This dynamometer features an adjustable grip span ranging from 3.5 to seven cm and measures handgrip strength in kilograms with a precision of 0.1 kg. IOP was measured using a portable rebound tonometer (Icare IC200, Tiolat Oy, Helsinki, Finland). This instrument, which has been clinically validated, has demonstrated a high level of agreement with Goldmann applanation tonometry, the gold standard for IOP measurement (Hackett et al., 2024). The Icare tonometer is advantageous due to its ease of use and ability to quickly acquire IOP readings without the need for topical anesthesia. Following the manufacturer’s instructions, participants were instructed to fixate on a distant target while six consecutive measurements were taken against the central cornea. The average of these six measurements was used for further analysis.

### Statistical analyses

Descriptive data are reported as means and SD. The normal distribution of the data was confirmed with the Shapiro–Wilk’s test (*p* > 0.05). Independent samples t tests were used to compare demographical information (age, body height, body mass, and body mass index) between POAG patients and healthy individuals. A three-factor mixed analysis of variance (ANOVA) with one within-subject factor (task intensity (maximal *vs.* submaximal)) and two between-subjects factors (group (POAG patients *vs.* healthy individuals) and sex (men *vs.* women)) was applied to handgrip strength values. Another four-factor mixed ANOVA with two within-subject factors (task intensity (maximal *vs.* submaximal) and time of measurement (pre *vs.* during *vs.* post)) and two between-subjects factors (group (POAG patients *vs.* healthy individuals) and sex (men *vs.* women)) was applied to IOP values. However, given that the main effect of sex (*p* = 0.699) as well as its different interactions (*p* ranged from 0.322 to 0.781) never reached statistical significance for IOP, the results section was simplified by showing the outputs of a three-factor mixed ANOVA without considering the factor sex. The Greenhouse-Geisser correction was applied when the Mauchly’s test revealed that sphericity assumptions were not met (*p* < 0.05). Pairwise comparisons were elucidated using Bonferroni *post-hoc* corrections. All statistical analyses were conducted using SPSS software version 25.0 (IBM Corp., Armonk, NY, USA). Statistical significance was accepted at an alpha level of 0.05.

## Results

No significant differences in age (*p* = 0.870), body mass (*p* = 0.861), body height (*p* = 0.718) or body mass index (*p* = 0.651) were observed between POAG patients and healthy individuals (Table 1).

**Figure 1:**
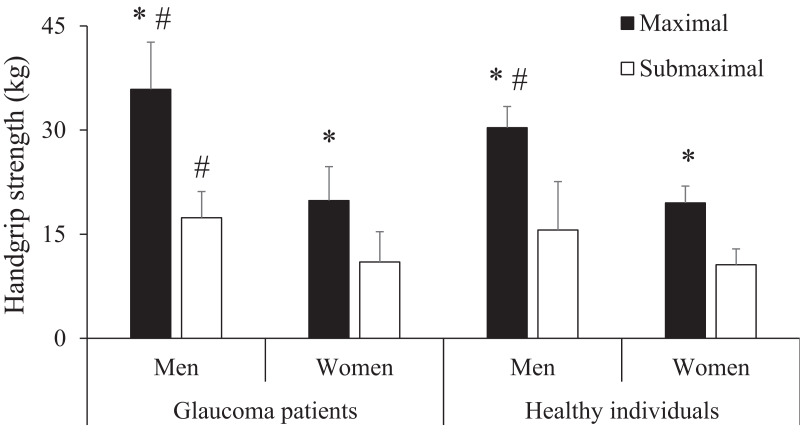
Comparison of isometric handgrip strength between the groups (glaucoma patients *vs.* healthy individuals), sex (men *vs.* women) and tasks (submaximal *vs.* maximal). *, significantly greater than the submaximal task; #, significantly greater than women. No significant differences between glaucoma patients and healthy individuals were observed for any of the four comparisons (*p* ranged from 0.066 to 0.828). Statistical significance was set at *p* < 0.05.

The main effect of task intensity reached statistical significance (*F* = 296.8, *p* < 0.001) due to the greater force values produced during the maximal task (24.8 ± 8.1 kg) compared to the submaximal task (13.0 ± 5.1 kg; *p* < 0.001). The main effect of sex also reached statistical significance (*F* = 58.9, *p* < 0.001) due to the greater force values produced by men (24.8 ± 8.1 kg) compared to women (13.0 ± 5.1 kg; *p* < 0.001). However, the main effect of group did not reach statistical significance (*F* = 2.6, *p* = 0.117). The interaction task intensity × sex reached statistical significance (*F* = 27.3, *p* < 0.001) because the differences between men and women were greater for the maximal task (68.1%) compared to the submaximal task (52.7%). However, the remaining interactions did not reach statistical significance (task intensity × group (*F* = 1.6, *p* = 0.220), group × sex (*F* = 2.6, *p* = 0.117), and task intensity × group × sex (*F* = 1.6, *p* = 0.210)). Pairwise comparisons are depicted in Fig. 1.

**Figure 2:**
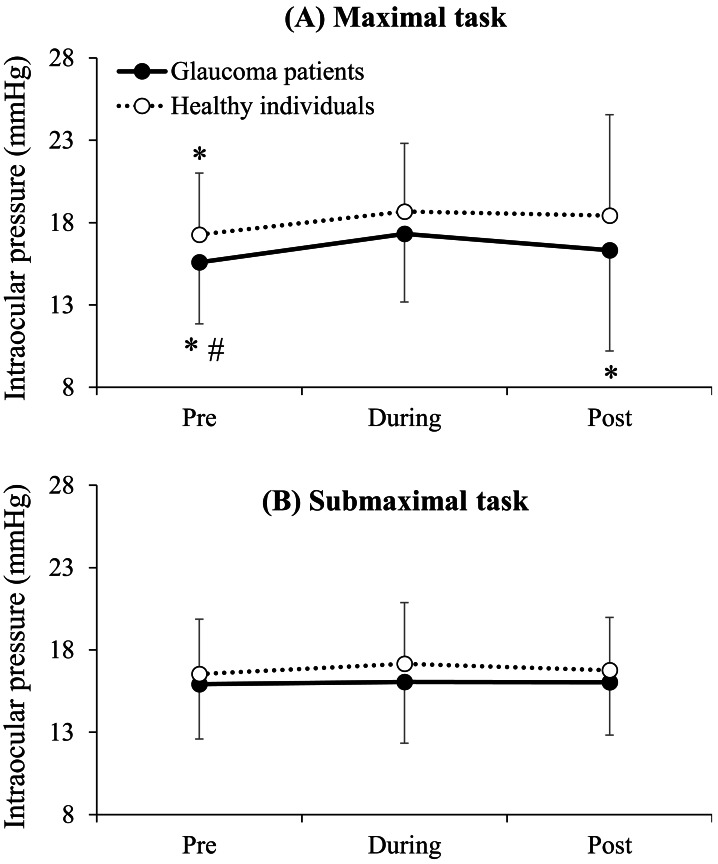
Comparison of intraocular pressure between the groups and times of measurement separately for the maximal (A) and submaximal (B) isometric handgrip strength tasks. *, significantly lower than during; #, significantly lower than post. No significant differences between glaucoma and normal groups were observed for any of the six comparisons (*p* ranged from 0.118 to 0.538). Statistical significance was set at *p* < 0.05.

The main effect of time reached statistical significance (*F* = 5.4, *p* = 0.016) due to the greater IOP values during the task (17.3 ± 3.7 mmHg) compared to pre (16.3 ± 3.2 mmHg: *p* < 0.001), but IOP values at post (16.9 ± 4.1 mmHg) were not significantly different compared to pre (*p* = 0.334) or during the task (*p* = 0.727). The main effect of task intensity also reached statistical significance (*F* = 13.3, *p* = 0.001) due to the greater IOP values for the maximal task (17.2 ± 4.1 mmHg) compared to the submaximal task (16.4 ± 3.2 mmHg: *p* = 0.001). The main effect of group (*F* = 1.5, *p* = 0.228) and none of the interactions reached statistical significance (task intensity × group (*F* = 3.7, *p* = 0.063), task intensity × time (*F* = 2.2, *p* = 0.140), group × time (*F* = 0.1, *p* = 0.903), and task intensity × group × time (*F* = 0.5, *p* = 0.601)). Pairwise comparisons are depicted in Fig. 2.

## Discussion

This study provides valuable insights into the IOP responsiveness of POAG patients to maximal and submaximal isometric handgrip strength tasks. Our findings reveal that IOP responses are comparable between POAG patients treated with hypotensive eye drops and healthy individuals, as the main effect of group and its interactions did not reach statistical significance. Notably, greater IOP values were observed for the maximal task compared to the submaximal task, regardless of group or gender. These results highlight that POAG patients treated with hypotensive eye drops show a similar IOP response to those observed in healthy individuals. Consequently, exercise prescriptions do not need to be adjusted based on gender or glaucoma condition (when medically controlled), though attention should be given to task intensity due to the greater IOP increases observed during maximal efforts.

Physical exercise, particularly resistance training, is essential for optimizing physical function across all ages (Garber et al., 2011). Despite its benefits, resistance training has sometimes been discouraged for glaucoma patients due to the potential for inducing acute IOP spikes (Bakke, Hisdal & Semb, 2009; Ezhilnila et al., 2021; Gene et al., 2023). However, not all resistance training is equal, as the manipulation of various components of the training stimulus can modulate IOP responses (Hackett et al., 2024). For instance, highly demanding strength training activities, such as lifting heavy loads or terminating sets close to muscular failure, have been shown to cause significant increases in IOP (Hackett et al., 2024). The most significant increases in IOP are likely observed during extenuating isometric exercises, with increments sometimes exceeding 20 mmHg (Vera et al., 2019). However, isometric handgrip exercises have been shown to produce lower increments in IOP (0.7–3, six mmHg) compared to other isometric exercises such as squats (7.5–8, eight mmHg), biceps curl (3.1–9.4 mmHg) and bench press (13–28 mmHg) (Dickerman et al., 1999; Vera et al., 2019), c) in healthy individuals without glaucoma. Therefore, it is prudent to first compare IOP responses to physical exercise between POAG patients and healthy individuals using an isometric task known to induce only moderate IOP variations.

Supporting our primary hypothesis, the IOP response to both maximal and submaximal isometric handgrip strength tasks was comparable for POAG patients and healthy controls. This is a novel finding, as no previous study has compared IOP responses to isometric efforts between these populations. A strength of our experimental protocol was the efficient matching of subjects in both groups by age, sex, and maximal handgrip strength, allowing for a more accurate comparison of IOP responses. The increase in IOP observed during the maximal isometric hand grip strength task in our study was less than that reported in healthy young subjects who compared IOP during and after exercise in women and men as a function of dominant hand (Pérez-Castilla et al., 2021). This difference could be potentially explained by the greater force values or lower age of the subjects (Pérez-Castilla et al., 2021). However, age seems to be a more plausible explanation, as no significant differences in IOP changes were observed between men and women in our study, despite men exerting more force during the isometric task. By directly comparing IOP responses between POAG patients and healthy individuals during a strength task known to induce IOP increments, our study suggests that with proper management, isometric training can be safely included in the exercise regimens of glaucoma patients.

The maximal, but not the submaximal, isometric handgrip strength task induced a significant increase in IOP during the task compared to pre-exercise. These results align with previous studies that have shown greater IOP increments with increased force demands. For example, some studies reported that performing isometric exercise for 2 min to the point of muscle failure causes an immediate and sustained increase in intraocular pressure in healthy adults (Vera et al., 2019). However, it is important to note that despite men producing approximately twice the force compared to women, the IOP response to exercise was comparable for both sexes. This suggests that IOP changes are determined not by absolute force values but by the force produced relative to the subject’s maximal capacity. Therefore, the importance of possessing high levels of muscle strength in daily activities like carrying a bag in the supermarket will be less demanding for stronger individuals, potentially mitigating IOP increases. Studies showing lower IOP increases during physical exercises in fitter individuals further support this recommendation (Vera et al., 2018a; Vera et al., 2018b). Therefore, while our results suggest that maximal intensity tasks should be avoided to mitigate IOP peaks during exercise, they also indicate that glaucoma patients can benefit from moderate intensity resistance training to increase strength capacity, which may play a protective role in preventing IOP spikes during daily activities.

While this study provides valuable insights into the comparative IOP responses during a common isometric task between POAG patients and healthy individuals, several limitations should be considered when interpreting our results. To prevent elevated IOP peaks in glaucoma patients, we chose the handgrip task because it involves a smaller muscle mass, and previous research has shown that exercises involving larger muscle groups can lead to greater increases in IOP (Vera et al., 2023). However, future studies should include exercises that involve larger muscle groups, such as squats and bench presses, as these are more commonly used in resistance training programs to increase muscle strength capacity. Also, the glaucoma population included in this study was diagnosed of POAG (*i.e.,* the most common type of glaucoma), and it is known that the mechanisms of outflow regulation would differ in other types of glaucoma such as angle-closure glaucoma, pigmentary glaucoma or pseudoexfoliative glaucoma (Braunger, Fuchshofer & Tamm, 2015). In the same line, POAG patients were treated with prostaglandin analogues alone or a combination of prostaglandin analogues and beta-blockers, and the mechanisms of action of those pharmacological strategies for IOP control are known to be different (Stamer & Acott, 2012). Further investigation is required in this regard. Lastly, POAG patients were medically treated and glaucoma progression was considered clinically stable, being plausible that subjects with ocular hypertension, undiagnosed individuals or glaucoma patients who are not medically stable can exhibit a more accentuated IOP responsiveness to the execution of resistance exercise as it has been shown for other provocative tests (Susanna Jr et al., 2017). These limitations should guide future research to improve external validity and understanding of the relationships between physical effort and IOP in glaucoma patients.

## Conclusions

This study found that IOP during maximal isometric handgrip strength tasks was moderate and did not differ significantly between POAG patients and healthy controls. In contrast, submaximal isometric handgrip tasks (performed at ∼50% of incorporating submaximal isometric strength exercises into the physical activity programs of POAG patients. Future research should assess the IOP response to resistance exercises involving larger muscle groups and higher physiological demand. Overall, this study highlights the potential role of appropriately dosed strength training in the multidisciplinary management of glaucoma.

##  Supplemental Information

10.7717/peerj.19845/supp-1 Supplemental Information 1 Rawdata

10.7717/peerj.19845/supp-2 Supplemental Information 2 Codebook

10.7717/peerj.19845/supp-3 Supplemental Information 3 STROBE Checklist

## Additional Information and Declarations

### Data Availability

The following information was supplied regarding data availability: Raw data is available in the Supplemental Files.
