# Supplementary material for: Intraocular pressure responses during maximal and submaximal handgrip strength tasks in primary open-angle glaucoma patients and healthy individuals
Source: PeerJ. 2025 Aug 26;13:e19845. doi: 10.7717/peerj.19845 (PMC12396205; doi:10.7717/peerj.19845)
Supplement: Supplemental Information 2 [file peerj-13-19845-s002.docx]

MaxStrength = Handgrip test maximal effort

MedStrength = Handgrip test medium effort

RightHand = Handgrip test performed with right hand

LeftHand = Handgrip test performed with left hand

RighEye = IOP measurement in right eye

LeftEye = IOP measurement in left eye

IOPBefore = IOP measurement before handgrip test

IOPDuring = IOP measurement during handgrip test

IOPPost = IOP measurement after handgrip test

Sex (Male) = 1

Sex (Female) = 2
